# Functional Fc Gamma Receptor Gene Polymorphisms and Long-Term Kidney Allograft Survival

**DOI:** 10.3389/fimmu.2021.724331

**Published:** 2021-08-23

**Authors:** Markus Wahrmann, Bernd Döhler, Marie-Luise Arnold, Sabine Scherer, Katharina A. Mayer, Susanne Haindl, Helmuth Haslacher, Georg A. Böhmig, Caner Süsal

**Affiliations:** ^1^Division of Nephrology and Dialysis, Department of Medicine III, Medical University of Vienna, Vienna, Austria; ^2^Institute of Immunology, Heidelberg University Hospital, Heidelberg, Germany; ^3^Department of Internal Medicine 3, Institute for Clinical Immunology, Friedrich-Alexander University Erlangen-Nuremberg, Erlangen, Germany; ^4^Department of Laboratory Medicine, Medical University of Vienna, Vienna, Austria

**Keywords:** Fc gamma receptor, antibody-mediated rejection, kidney transplantation, anti-HLA antibodies, allograft survival

## Abstract

The functional Fc gamma receptor (FcγR) IIIA polymorphism *FCGR3A*-V/F158 was earlier suggested to determine the potential of donor-specific HLA antibodies to trigger microcirculation inflammation, a key lesion of antibody-mediated renal allograft rejection. Associations with long-term transplant outcomes, however, have not been evaluated to date. To clarify the impact of *FCGR3A*-V/F158 polymorphism on kidney transplant survival, we genotyped a cohort of 1,940 recipient/donor pairs. Analyzing 10-year death-censored allograft survival, we found no significant differences in relation to *FCGR3A*-V/F158. There was also no independent survival effect in a multivariable Cox model. Similarly, functional polymorphisms in two other activating FcγR, *FCGR2A*-H/R131 (FcγRIIA) and *FCGR3B*-NA1/NA2 (FcγRIIIB), were not associated with outcome. There were also no significant survival differences among patient subgroups at increased risk of rejection-related injury, such as pre-sensitized recipients (> 0% panel reactivity; n = 438) or recipients treated for rejection within the first year after transplantation (n = 229). Our study results suggest that the earlier shown association of FcγR polymorphism with microcirculation inflammation may not be strong enough to exert a meaningful effect on graft survival.

## Introduction

Antibody-mediated rejection (ABMR) is a major obstacle to long time transplant survival (1, 2). Alloantibodies occur as a result of previous transplants, blood transfusions, or pregnancies, or they are formed *de novo* after transplantation (3–5). Donor-specific antibodies against HLA antigens (DSA), which are predominantly of the IgG subtype, may exert their detrimental action *via* complement activation and/or antibody-dependent cell cytotoxicity (ADCC). The latter includes the binding of the antibody’s Fc domain to Fc receptors on a variety of different immune cells, such as natural killer (NK) cells, which are suspected to play a key role in rejection (6, 7). Signaling *via* activating Fc gamma receptors (FcγR) may initiate a variety of effector mechanisms, such as immune cell recruitment and activation, production and secretion of reactive oxygen species, cytotoxins and cytokines, all of which may contribute to allograft injury (8).

Several gene polymorphisms have been demonstrated to impact on the functionality of activating FcγR. A single nucleotide polymorphism (SNP) in the coding sequence of FcγRIIIA, which is expressed on the surface of monocytes/macrophages and natural killer (NK) cells, encodes a valine (V) to phenylalanine (F) amino acid substitution at position 158 (*FCGR3A*-V/F158) and is associated with decreased immune cell activation and altered binding characteristics to IgG1 and IgG3 (9, 10). Another functional polymorphism is located in the sequence of the myeloid cell receptor FcγRIIA. This SNP causes a transition from a histidine (H) to an arginine (R) at amino acid (aa) position 131 in the Ig-like domain (*FCGR2A*-H/R131), which is associated with decreased IgG2 binding and Fc-mediated phagocytosis (9, 11). Furthermore, the neutrophilic receptor FcγRIIIB harbors the so-called neutrophil antigen (NA) system, which consists of a group of one synonymous and four nonsynonymous mutations. These constitute two isoforms NA1 and NA2 consisting of 4 amino acid differences which result in different N-linked glycosylation and receptor function (12).

In the last decade, various studies have analyzed the clinical impact of individual functional FcγR polymorphisms in cohorts of transplant patients. Nevertheless, the true effect of such polymorphisms in this specific setting has remained ill-defined, and study results have been inconsistent. This may have its explanation in small sample size, differences in patient selection and characteristics, as well as endpoints studied (13–17).

In a recent study our group has investigated the role of functional FcγR polymorphisms in the specific context of DSA-triggered graft injury, hypothesizing a link between receptor functionality, the extent of microcirculation inflammation, and, as a possible consequence, renal allograft dysfunction and loss. Our study, which included 85 DSA-positive allograft recipients identified upon cross-sectional antibody screening of 745 kidney allograft recipients in outpatient care, revealed a significant association of FcγIIIA functional polymorphism V/F158 with peritubular capillaritis (ptc), a characteristic feature of ABMR. In contrast, no such associations were found for functional variants of FcγRIIA and FcγRIIIB. There were no associations with renal functional course or graft loss rate, however, the study was not powered to detect subtle differences in renal allograft outcomes (18). Analyzing a cohort of 133 chronic active ABMR patients, Litjens et al. (19) confirmed our finding of an association of FcγIIIA functional polymorphism V/F158 with morphologic features of microcirculation inflammation, and, in an experimental model, with NK cell activation. In some contrast to our study, however, they found a significant survival effect, a finding that needs to be validated by an adequately powered study design (19).

Considering the presumed critical role of FcγIIIA-triggered NK cell activation in ABMR and the recent observation of an association of FcγIIIA polymorphisms with the extent of DSA-triggered microcirculation inflammation, we hypothesized a potential impact of FcγRIIIA functionality on clinically relevant endpoints. Our present study was primarily designed to clarify the impact of functional polymorphisms of low affinity Fcγ receptor FcγRIIIA (and, in addition, FcγRIIA and IIIB) on long-term graft survival in a large cohort study powered to detect meaningful outcome differences. The study included approximately 2,000 donor-recipient pairs derived from the international Collaborative Transplant Study (CTS). Included patients were transplanted during a period from 1988 to 2006, which allowed for a long-term clinical follow-up of 10 years.

## Materials and Methods

### Study Design and Participants

To assess the impact of functional FcγR polymorphisms on long-term transplant survival we analyzed a large cohort derived from the Collaborative Transplant Study (CTS, www.ctstransplant.org). FcγR genotyping (Medical University of Vienna) and statistical data analysis (University of Heidelberg) were carried out in a blinded fashion.

Biologic material and clinical data used in this retrospective genetic study were collected prospectively, after written informed consent (approval by the local ethics committees of contributing transplantation centers in Europe and Northern America participating in the CTS). The present retrospective genotyping study was approved under the application number 083/2005 of the ethics committee of the University of Heidelberg.

The criteria for inclusion of recipient and donor pairs were: (1) deceased donor kidney transplantation, (2) transplantation between 1988 and 2006 in Europe or Northern America; (3) single-organ transplantation; (4) Caucasian recipient and donor; (5) complete database record of original disease, recipient and donor age, graft number, or HLA typing; and (6) known immunosuppression scheme (intention to treat). Initially, 2,070 recipient-donor pairs were randomly chosen from the DNA bank of the CTS study. For 3,880 of the selected samples, sufficient DNA was available, and complete Fc receptor genotyping was possible for both recipient and donor, resulting in a total of 1,940 transplants from 52 transplant centers in 13 countries, which were finally included. There was no pairing of one donor with two recipients. As shown in **Supplemental Figure S1**, comparison of graft survival between selected patients and 26,736 recipients who would have been eligible but were not genotyped, showed no difference in overall graft survival (P = 0.85), indicating an indeed random and representative sample selection. With approximately 2,000 recipients and a significance level of 0.05, this study has a test power of 80% to detect a relative risk of ≤ 0.8 or ≥ 1.25.

### Fc Receptor Genotyping

Genomic DNA was purified from peripheral blood of recipients and from lymph node or spleen samples of donors. Analysis of two exon polymorphisms, *FCGR3A*-V/F158 (denoted as rs396991) and *FCGR2A*-H/R131 (rs1801274) and one intron polymorphism associated with *FCGR3B*-NA1/NA2 (rs35139848) was carried out in 384-well optical plates on a 7900HT fast real-time PCR system (Applied Biosystems, Rotkreuz, Switzerland) using TaqMan^®^ SNP Genotyping Assay and TaqMan^®^ Universal PCR Master Mix (Fisher Scientific Austria). According to a previously published protocol, two TaqMan^®^ MGB probes were used for each genotyping assay. The first probe detecting allele 1 (Histidine/*FCGR2A*, Valine/*FCGR3A* and NA1/*FCGR3B*) was labelled with VIC^®^ dye, and the second with FAM™ labelled probe was detecting allele 2 (Arginine/*FCGR2A*, Phenylalanine/*FCGR3A* or NA2/*FCGR3B*) (18).

### Statistical Analysis

Continuous data were presented as mean and standard deviation (SD), and categorical variables as absolute and relative frequencies. Chi-quadrat tests were used to compare categorical data. Kruskal-Wallis tests were applied for continuous variables. For all genotype-related statistical analyses patient numbers were high enough to divide homozygotes and heterozygotes into three groups; combining a low-numbered homozygous group with heterozygotes was not necessary. Kaplan-Meier analysis was used for calculation of 10-year graft survival. The Mantel-Cox log-rank test was applied for the comparison of survival between groups. Multivariable Cox regression analyses with the variables year of transplantation, geographical region (continent), first or re-transplant, recipient and donor sex and age, cold ischemia time, number of HLA A+B+DR mismatches, preformed panel-reactive antibodies, original disease leading to end stage renal failure, and immunosuppressive therapy (intention to treat) was performed to eliminate the influence of possible confounders. Hazard ratios (HR) are presented with 95% confidence intervals (CI). A two-sided P value less than 0.05 was considered statistically significant. For statistical analysis IBM SPSS Statistics (version 25, SPSS Inc., Chicago, IL, USA) was used. The R package “hwde” was used for exact testing of Hardy-Weinberg equilibrium (20).

## Results

### Patient Characteristics and FcγR Genotyping Results

This retrospective cohort study included donor-recipient pairs of 1,940 deceased donor renal transplantations performed between 1988 and 2006 and reported to the CTS. For included patients, genotyping of genomic DNA samples of three pre-specified FCGR gene loci was performed: *FCGR3A*-V/F158 (denoted as rs396991), *FCGR2A*-H/R131 (rs1801274) and *FCGR3B*-NA1/NA2 (rs35139848), respectively. Baseline characteristics of the overall cohort stratified by FcγR genotypes are provided in **Table 1** as well as in **Supplemental Tables S1, S2**. All study subjects were Caucasian with a geographic origin in Europe (89%) or Northern America (11%). The majority of patients (n = 1,681, 87%) were recipients of a first kidney transplant. Of note, 438 recipients (24%) had detectable panel-reactive antibodies before transplantation. 1,676 recipients had a functioning graft with documented serum creatinine at year 1 after transplantation, and of these, the information of rejection during the first year after transplantation is known for 1,010 (60%) patients. Ninety-five percent of included subjects received initial immunosuppression with a calcineurin inhibitor, either cyclosporine A (79%) or tacrolimus (16%). In addition, 47% of recipients received azathioprine, and 33% mycophenolic acid, respectively. Among studied patients, 34% were given antibody induction (IL-2 receptor antibody, 8%; depleting anti-lymphocyte antibody, 26%) (**Table 1**). The allele frequency of variant F158 of the *FCGR3A*-V/F158 locus was 64.0%, that of the donor 64.3%, and frequencies of variant H131 of the *FCGR2A*-H/R131 polymorphism was 53.7% and 52.7% (donors). The allele frequency of the NA2 variant of the *FCGR3B* locus was 64.5% in recipients and 65.4% in donors. Genotype frequencies did not deviate from Hardy-Weinberg equilibrium (**Supplemental Table S3**).

**Table 1:** Patient baseline characteristics in relation to *FCGR3A*-V/F_158_ genotype.

Characteristic	All Patients	F/F_158_	V/F_158_	V/V_158_	*P* value
	(n = 1,940)	(n = 799)	(n = 884)	(n = 257)	
Female recipient sex, n (%)	762 (39)	292 (37)	358 (40)	110 (43)	0.11
Recipient age, mean ± SD (years)	46.8 ± 13.7	46.8 ± 13.6	46.3 ± 13.8	48.9 ± 13.3	0.021
Geographic origin, n (%)					0.57
Europe	1,729 (89)	706 (88)	795 (90)	228 (89)	
Northern America	211 (11)	93 (12)	89 (10)	29 (11)	
First renal allograft, n (%)	1,681 (87)	704 (88)	758 (86)	219 (85)	0.28
Underlying renal disease, n (%)					0.78
Glomerulonephritis	626 (32)	253 (32)	282 (32)	91 (35)	
Polycystic kidneys	241 (12)	94 (12)	114 (13)	34 (13)	
Diabetes mellitus	174 (9)	78 (10)	74 (8)	22 (9)	
Other	899 (46)	374 (47)	414 (47)	110 (43)	
Donor sex, n (%)					0.50
Female	768 (40)	308 (39)	350 (40)	110 (43)	
Male	1,169 (60)	488 (61)	534 (60)	147 (57)	
Donor age, mean ± SD (years)	40.4 ± 16.7	39.9 ± 16.7	40.7 ± 16.6	41.2 ± 16.8	0.58
Cold ischemia time, mean ± SD (hours)	20.3 ± 8.0	20.2 ± 7.8	20.3 ± 8.1	20.4 ± 8.5	0.78
HLA A+B+DR mismatches, n (%)					0.87
0 – 1	227 (12)	98 (12)	103 (12)	26 (10)	
2 – 4	1,440 (74)	585 (73)	659 (75)	196 (76)	
5 – 6	273 (14)	116 (15)	122 (14)	35 (14)	
Panel-reactive antibodies, n (%)					0.23
= 0%	1,370 (76)	581 (78)	608 (74)	181 (75)	
> 0%	438 (24)	166 (22)	213 (26)	59 (25)	
Initial immunosuppression, n (%)					0.55
Cyclosporine A	1,540 (79)	638 (80)	707 (80)	195 (76)	
Tacrolimus	304 (16)	126 (16)	131 (15)	47 (18)	
No calcineurin inhibitor	96 (5)	35 (4)	46 (5)	15 (6)	
Azathioprine	903 (47)	379 (47)	417 (47)	107 (42)	0.44
Mycophenolic acid	648 (33)	256 (32)	299 (34)	93 (36)	
No antimetabolite agent	389 (20)	164 (21)	168 (19)	57 (22)	
Induction therapy, n (%)					0.45
IL-2R antibody	161 (8)	65 (9)	69 (8)	27 (11)	
Depleting anti-lymphocyte agent	499 (26)	164 (22)	201 (24)	54 (22)	
Without	1,252 (65)	533 (70)	559 (67)	160 (66)	

F, phenylalanine; V, valine; SD, standard deviation; HLA, human leukocyte antigen; IL-2R, interleukin 2 receptor.

### Impact of Recipient FcγR Polymorphisms on Renal Transplantation Outcomes

**Figure 1:**
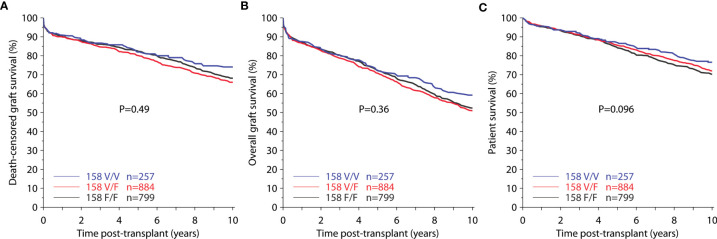
Death-censored graft survival **(A)**, overall graft survival **(B)**, and patient survival **(C)** for genotypic groups of *FCGR3A*-V/F158 (total 1,940 recipients).

Kaplan Meier analyses revealed no significant differences of death-censored allograft survival, overall graft survival, or patient survival in relation to *FCGR3A*-V/F158 genotype (**Figure 1**).

**Figure 2:**
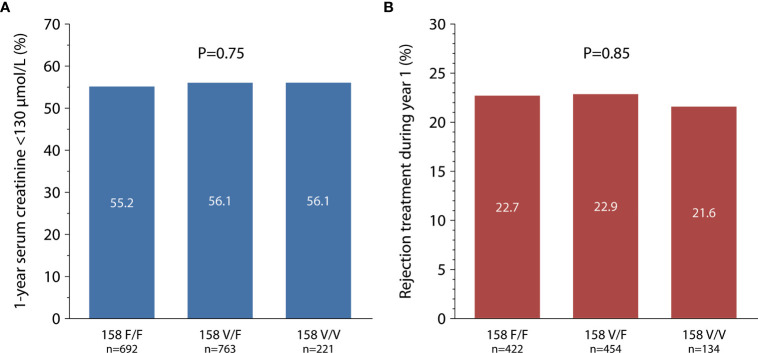
*FCGR3A*-V/F_158_ polymorphism in relation to 1-year serum creatinine [**(A)** total 1,676 recipients] and treated rejection episodes within the first year after transplantation [**(B)** total 1,010 recipients].

In a multivariable Cox regression analysis, the following possible confounders were included: year of transplantation, geographical region (continent), first or re-transplant, recipient and donor sex and age, cold ischemia time, number of HLA A+B+DR mismatches, pre-formed panel-reactive antibodies, original disease leading to end stage renal failure, and immunosuppressive therapy (intention to treat). The hazard ratio (HR) for death-censored graft loss during the first 10 years post-transplant was with 1.09 [95% confidence interval (95% CI) 0.91 – 1.31, P = 0.36] for heterozygous V/F158 recipients and 0.84 (95% CI 0.63 – 1.13, P = 0.25) for homozygous V/V158 recipients not significantly different from the homozygous reference group of F/F158 recipients. Furthemore, the *FCGR3A*-V/F158 polymorphism was not associated with impaired allograft function or increased need for rejection treatments within the first year after transplantation (P = 0.75 and 0.85, respectively; **Figure 2**).

Similarly, analyses of *FCGR2A*-H/R131 and *FCGR3B*-NA1/NA2 genotypes did not reveal any impact on death-censored graft survival (**Supplemental Figure S2**), graft function or requirement of rejection treatment (**Supplemental Figure S3**).

### Impact of FcγR Polymorphisms on Transplant Outcome in Presensitized Patients

**Figure 3:**
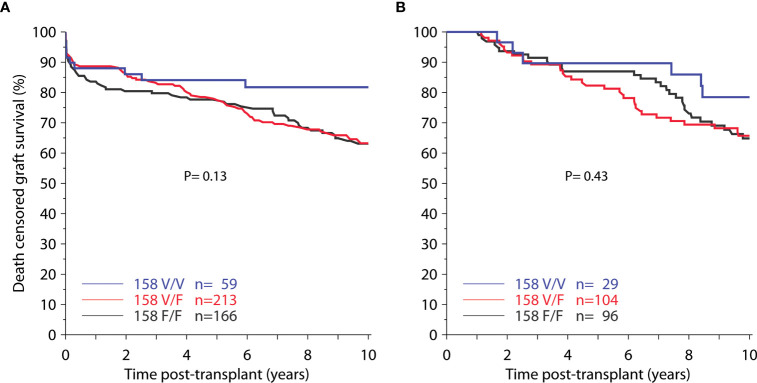
Death-censored graft survival among **(A)** 438 pre-sensitized recipients (panel reactivity > 0%) and **(B)** 229 recipients with treated rejection during the first-post-transplant year, in relation to *FCGR3A*-V/F158 genotype.

To evaluate the impact of FcγR polymorphisms in patients at risk of DSA-triggered graft injury, we focused on 438 recipients with panel reactive antibodies (PRA > 0%) at the time of transplantation. Despite their undefined DSA status, such patients are known to have a higher risk of developing ABMR and death-censored graft survival (21). In Kaplan-Meier analyses, however, we observed no associations of FcγRIIIA, FcγRIIA and FcγRIIIB polymorphisms with death-censored 10-year graft survival (**Figure 3A** and **Supplemental Figure S4A**). The hazard ratio for death-censored graft loss during the first 10 years post-transplant was 0.89 (CI 0.62 – 1.29, P = 0.55) for heterozygous V/F158 recipients and 0.62 (CI 0.31 – 1.23, P = 0.17) for homozygous V/V158 recipients and thus not significantly different from the homozygous reference group of F/F158 recipients.

### Impact of FcγR Polymorphisms on Transplant Outcome in Patients With Allograft Rejection

Further sub-analyses focused on 229 patients who were treated for rejection during the first year. Again, there were no statistically significant associations of FcγRIIIA, FcγRIIA and FcγRIIIB polymorphisms with death-censored 10-year graft survival (**Figure 3B** and **Supplemental Figure S4B**).

### Impact of ‘Donor’ FcγR Polymorphisms on Transplant Outcome

**Figure 4:**
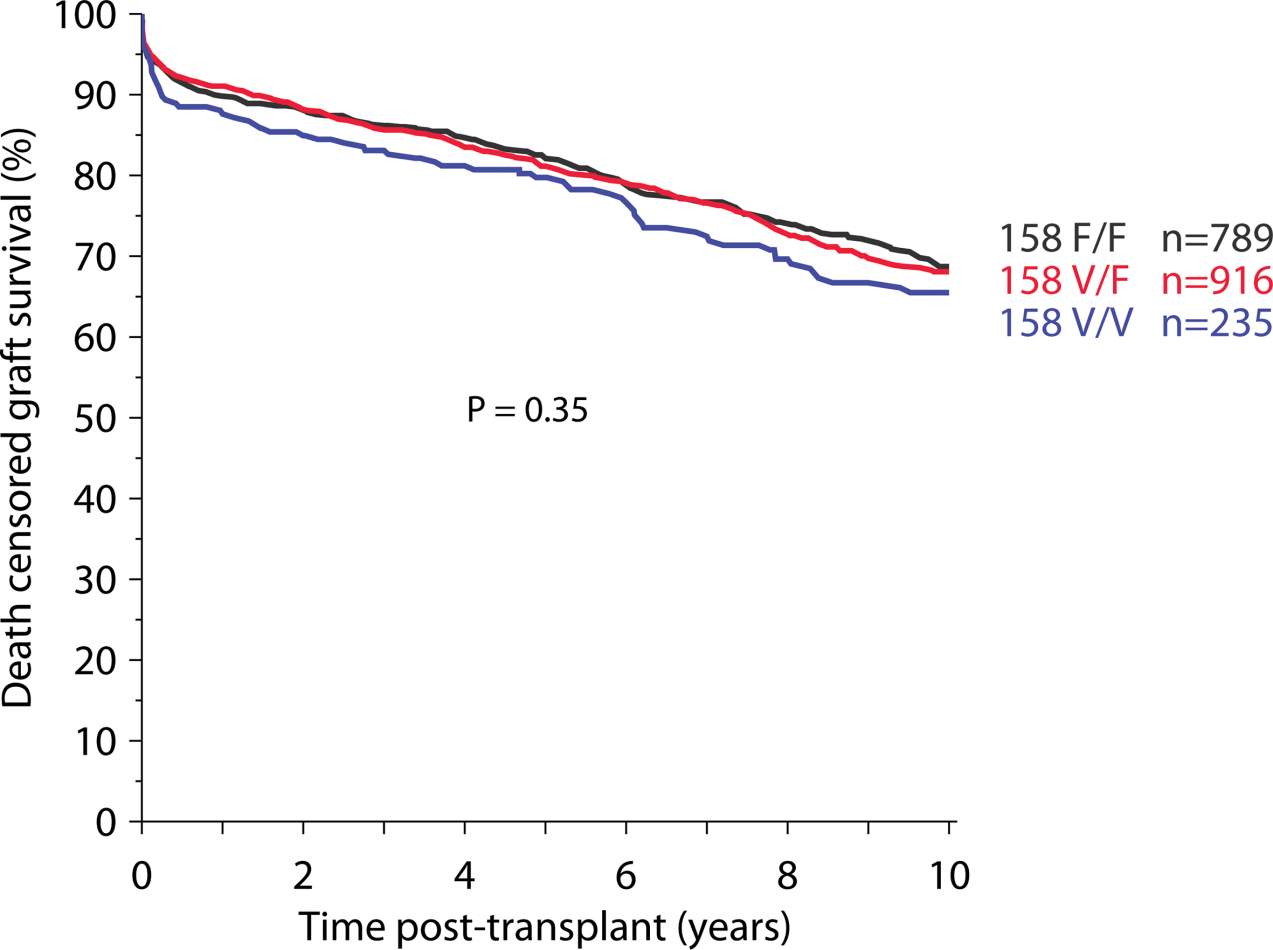
Impact of donor-derived FcγRIIIA polymorphism on death-censored graft survival (total 1,940 recipients).

Finally, we evaluated the association of ‘donor’ FcγR genotype status with 10-year death-censored graft survival. Again, we divided donor subjects into three groups of the respective FcγR locus. There was no difference in 10-year death-censored graft survival between the donor genotype groups in any of the three loci *FCGR3A*-V/F158, *FCGR2A*-H/R131 and *FCGR3B*-NA1/NA2 (**Figure 4** and **Supplemental Figure S5**).

## Discussion

In this large retrospective study of approximately 2,000 kidney transplant recipients, we investigated whether recipient or donor FcγR gene variants have an impact on long-term allograft survival. However, analyzing death-censored graft survival, there was no association of the function-determining polymorphisms in any of the three receptors FcγRIIIA, FcγRIIA and FcγRIIIB, neither in recipients nor in donors, on 10-year survival. Furthermore, we did not observe any association of recipient FcγR polymorphisms with other clinical endpoints, such as graft function or treatment for rejection within the first year after transplantation. Hypothesizing that survival differences might only be visible in patients with antibody-mediated rejection, we selected patients whose data availability indicated that they were at increased risk for this type of rejection: recipients with anti-HLA antibodies at the time of transplantation or rejection treatment within the first post-transplant year. However, also in these sub-analyses associations of FcγR polymorphisms with transplant outcome were not observed.

In an earlier study of 85 long-term kidney transplanted DSA-positive patients, we found that recipients with at least one allele of the high-affinity variant of the *FCGR3A* locus (V/V158 or V/F158) had a greater extent of ptc than homozygous carriers of the low-affinity allele F158 (18). Peritubular capillaritis is a major constituent of antibody-mediated rejection, associated with inferior long-term graft outcomes (22, 23). *In vitro* experiments showed that a NK cell line, homozygously expressing *FCGR3A*-V158, produced at least two times more interferon gamma (IFN-γ) upon incubation with anti-HLA antibody-spiked T cells than the same cell line transfected with the homozygous F158 variant (18). More recently, NK cell activation and IFN-γ-triggered chemokine expression were suggested to critically contribute to transplant injury in ABMR (6, 7, 24, 25). However, in the same study we failed to demonstrate a negative impact of high functional receptor 3A variant (nor those of any other FcγR polymorphism) on graft function or on 2-year allograft survival (18). Since this earlier study was not primarily designed to determine small differences in survival rates, we initiated the present large-scale study with a follow-up period of 10 years, the results of which point in the same direction, namely no association of FcγR polymorphisms with kidney allograft survival.

At first sight our results may be in some contrast to other studies: for instance, a recent lung transplant study identified the V/V158 genotype of FcγRIIIA as an independent susceptibility factor associated with higher rates of acute rejection in the first three months after transplantation (26). An even more recent for-cause biopsy-based study by Litjens et al. (19) revealed no different frequency of *FCGR3A*-V/F158 variants comparing chronic active ABMR patients and control kidney transplant recipients. However, this study suggested associations of genotype V/V158 with a higher degree of glomerulitis (g) (but not ptc) and with inferior 3-year graft survival after diagnosis. Yuan et al. and Ozkayin et al. reported a significant shift towards a higher allele frequency of FcγRIIA R131 in acute rejectors (15, 16) and Arnold et al. (17) demonstrated a significantly shorter graft survival of homozygous carriers of R131, an effect that was even more pronounced when HLA antibodies were present. However, all of these aforementioned studies have in common that their case numbers are low, while our present study relies on 1,940 transplant patients.

However, our large registry study has some inherent limitations. These may include the lack of granular pre- and post-transplant HLA antibody data as well as detailed data regarding the type of treated rejections, including a clear-cut differentiation between TCMR and ABMR, or individual rejection sub-phenotypes or single criteria. Prior to the year 2000, ABMR criteria in the Banff scheme and solid phase single bead HLA antibody analysis were not established, and, accordingly, all this exact information was not available. The pre-transplant presence of HLA antibodies was documented in the CTS as lymphocytotoxic PRA without differentiation between DSA and non-DSA. We are aware that in our subanalysis of 438 sensitized recipients, a considerable proportion of patients may have not been exposed to DSA, either before or after transplantation, and were thus not at risk of ADCC-triggered graft injury. Moreover, one may argue, that only a minor proportion of patients treated for rejection (second sub-analysis of 229 recipients) actually had DSA-triggered ABMR, and a majority of them may have had TCMR without any preformed or *de novo* DSA.

We have found previously an interrelation between high-affinity FcγRIIIA V158 and ptc in a cohort of 85 DSA-positive long-time transplanted patients (comprising 49 subclinical ABMR), however, we were not able to demonstrate an impact of FcγRIIIA V/F158 polymorphism (or of any of the other function-defining polymorphisms in the other two receptors) on 2-year allograft survival in the same study (18).

In summary of this present register study we confirmed this previous finding and found no impact of FcγRIIIA V/F158 polymorphism (or of any of the other function-defining polymorphisms in the other two receptors) on 10-year allograft survival, neither in the large cohort of 1,940 recipients nor in our two subanalysis cohorts of 438 pre-transplant anti-HLA antibody-positive patients and 229 patients with rejection treatment during the first post-transplant year.

Based on our current results, we believe that FcγR genotyping may have limited relevance for unselected transplant cohorts. Future studies, however, will have to clarify its usefulness for risk stratification in selected cohorts of patients, e.g. pre- or post-transplant DSA-positive subjects who are at particular risk of ABMR.

## Data Availability Statement

The original contributions presented in the study are included in the article/**Supplementary Material**. Further inquiries can be directed to the corresponding author.

## Ethics Statement

The studies involving human participants were reviewed and approved by Ethics Committee of the University of Heidelberg. The patients/participants provided their written informed consent to participate in this study.

## Author Contributions

Conceptualization, MW, BD, GB and CS. Methodology, M-LA, HH. Formal analysis, MW, SH. Investigation, MW, KM. Resources, HH. Data curation, BD, SS, CS. Writing-original draft preparation, MW, GB. Writing-review and editing, BD, KM. Visualization, BD. Supervision, CS. Funding acquisition, GB. All authors contributed to the article and approved the submitted version.

## Funding

This research was funded by the Austrian National Bank (to GB), grant number 12471).

## Conflict of Interest

The authors declare that the research was conducted in the absence of any commercial or financial relationships that could be construed as a potential conflict of interest.

## Publisher’s Note

All claims expressed in this article are solely those of the authors and do not necessarily represent those of their affiliated organizations, or those of the publisher, the editors and the reviewers. Any product that may be evaluated in this article, or claim that may be made by its manufacturer, is not guaranteed or endorsed by the publisher.
