# Supplementary material for: Functional Fc Gamma Receptor Gene Polymorphisms and Long-Term Kidney Allograft Survival
Source: Front Immunol. 2021 Aug 23;12:724331. doi: 10.3389/fimmu.2021.724331 (PMC8420807; doi:10.3389/fimmu.2021.724331)
Supplement: Supplementary file 1 [file DataSheet_1.docx]

Supplementary Material

# Supplementary Tables

**Supplemental Table S1.** Patient baseline characteristics in relation to FcγRIIA polymorphism.

| **Characteristic** | **All patients** (n = 1,940) | **R/R_131_** (n = 399) | **H/R_131_** (n = 999) | **H/H_131_** (n = 542) | ***P*** |
| --- | --- | --- | --- | --- | --- |
| Female recipient sex, n (%) | 762 (39) | 154 (39) | 385 (39) | 221 (41) | 0.67 |
| Recipient age, mean ± SD (years) | 46.8 ± 13.7 | 47.3 ± 13.5 | 46.7 ± 13.9 | 46.8 ± 13.6 | 0.83 |
| Geographic origin, n (%)  Europe  Northern America | 1,729 (89) 211 (11) | 359 (90) 40 (10) | 880 (88) 119 (12) | 490 (90) 52 (10) | 0.31 |
| First renal allograft, n (%) | 1,681 (87) | 338 (85) | 873 (87) | 470 (87) | 0.41 |
| Underlying renal disease, n (%)  Glomerulonephritis  Polycystic kidneys  Diabetes mellitus  Other | 626 (32) 241 (12) 174 (  9) 899 (46) | 124 (31) 48 (12) 47 (12) 180 (45) | 324 (32) 116 (12) 85 (  9) 474 (47) | 178 (33) 78 (14) 42 (  8) 244 (45) | 0.28 |
| Donor sex, n (%)  Female  Male | 768 (40) 1,169 (60) | 139 (35) 260 (65) | 407 (41) 590 (59) | 222 (41) 319 (59) | 0.088 |
| Donor age, mean ± SD (years) | 40.4 ± 16.7 | 40.6 ± 16.3 | 39.9 ± 17.0 | 41.4 ± 16.3 | 0.27 |
| Cold ischemia time, mean ± SD (hours) | 20.3 ± 8.0 | 21.1 ± 7.9 | 19.9 ± 7.6 | 20.3 ± 8.9 | 0.041 |
| HLA A+B+DR mismatches, n (%)  0 – 1  2 – 4  5 – 6 | 227 (12) 1,440 (74) 273 (14) | 50 (13) 301 (75) 48 (12) | 125 (13) 735 (74) 139 (14) | 52 (10) 404 (74) 86 (16) | 0.25 |
| Panel-reactive antibodies, n (%)  = 0%  > 0% | 1,370 (76) 438 (24) | 284 (77) 85 (23) | 701 (75) 235 (25) | 385 (77) 118 (23) | 0.66 |
| Initial immunosuppression, n (%)  Cyclosporine A  Tacrolimus  No calcineurin inhibitor | 1,540 (79) 304 (16) 96 (  5) | 323 (81) 60 (15) 16 (  4) | 783 (78) 160 (16) 56 (  6) | 434 (80) 84 (15) 24 (  4) | 0.67 |
| Azathioprine  Mycophenolic acid  No antimetabolite agent | 903 (47) 648 (33) 389 (20) | 191 (48) 122 (31) 86 (22) | 465 (47) 331 (33) 203 (20) | 247 (46) 195 (36) 100 (18) | 0.48 |
| Induction therapy, n (%)  IL-2R antibody  Depleting anti-lymphocyte agent  Without | 161 (  8) 499 (26) 1,252 (65) | 32 (  9) 99 (26) 245 (65) | 85 (  9) 209 (22) 648 (69) | 44 (  9) 111 (22) 359 (70) | 0.49 |

R, arginine; H, histidine; SD, standard deviation; HLA, human leukocyte antigen; IL-2R, interleukin 2 receptor

**Supplemental Table S2.** Patient baseline characteristics in relation to FcγRIIIB polymorphism.

| **Characteristic** | **All patients** (n = 1,940) | **NA2/NA2** (n = 816) | **NA1/NA2** (n = 872) | **NA1/NA1** (n = 252) | ***P*** |
| --- | --- | --- | --- | --- | --- |
| Female recipient sex, n (%) | 762 (39) | 325 (40) | 344 (39) | 91 (36) | 0.56 |
| Recipient age, mean ± SD (years) | 46.8 ± 13.7 | 47.4 ± 13.8 | 46.5 ± 13.5 | 46.3 ± 14.0 | 0.34 |
| Geographic origin, n (%)  Europe  Northern America | 1,729 (89) 211 (11) | 738 (90) 78 (10) | 771 (88) 101 (12) | 220 (87) 32 (13) | 0.25 |
| First renal allograft, n (%) | 1,681 (87) | 694 (85) | 770 (88) | 217 (86) | 0.14 |
| Underlying renal disease, n (%)  Glomerulonephritis  Polycystic kidneys  Diabetes mellitus  Other | 626 (32) 241 (12) 174 (  9) 899 (46) | 265 (32) 100 (12) 62 (  8) 389 (48) | 267 (31) 113 (13) 89 (10) 403 (46) | 94 (37) 29 (12) 23 (  9) 106 (42) | 0.27 |
| Donor sex, n (%)  Female  Male | 768 (40) 1,169 (60) | 319 (39) 497 (61) | 359 (41) 511 (59) | 90 (36) 161 (64) | 0.28 |
| Donor age, mean ± SD (years) | 40.4 ± 16.7 | 40.2 ± 16.7 | 41.1 ± 16.6 | 39.0 ± 16.8 | 0.21 |
| Cold ischemia time, mean ± SD (hours) | 20.3 ± 8.0 | 20.5 ± 7.8 | 20.0 ± 8.3 | 20.2 ± 7.8 | 0.30 |
| HLA A+B+DR mismatches, n (%)  0 – 1  2 – 4  5 – 6 | 227 (12) 1,440 (74) 273 (14) | 95 (12) 602 (74) 119 (15) | 108 (12) 652 (75) 112 (13) | 24 (10) 186 (74) 42 (17) | 0.44 |
| Panel-reactive antibodies, n (%)  = 0%  > 0% | 1,370 (76) 438 (24) | 578 (76) 183 (24) | 607 (75) 203 (25) | 185 (78) 52 (22) | 0.61 |
| Initial immunosuppression, n (%)  Cyclosporine A  Tacrolimus  No calcineurin inhibitor | 1,540 (79) 304 (16) 96 (  5) | 638 (78) 130 (16) 48 (  6) | 702 (81) 134 (15) 36 (  4) | 200 (79) 40 (16) 12 (  5) | 0.55 |
| Azathioprine  Mycophenolic acid  No antimetabolite agent | 903 (47) 648 (33) 389 (20) | 374 (46) 273 (33) 169 (21) | 415 (48) 284 (33) 173 (20) | 114 (45) 91 (36) 47 (19) | 0.82 |
| Induction therapy, n (%)  IL-2R antibody  Depleting anti-lymphocyte agent  Without | 161 (  8) 499 (26) 1,252 (65) | 74 (10) 186 (24) 505 (66) | 68 (  8) 185 (22) 575 (69) | 19 (  8) 48 (20) 172 (72) | 0.40 |

NA, neutrophil antigen; SD, standard deviation; HLA, human leukocyte antigen; IL-2RA, interleukin 2 receptor

**Supplemental Table S3.** Genotype frequencies of Fc gamma receptor polymorphisms.

| **Fc gamma receptor** | **Recipients** (n = 1,940) | **Donors** (n = 1,940) |
| --- | --- | --- |
| FcγIIIA, n (%) F/F_158_ V/F_158_ V/V_158_  *P* value of HWE | 799 (41) 884 (46) 257 (13)  0.62 | 789 (41) 916 (47) 235 (12)  0.24 |
| FcγIIA, n (%) R/R_131_ H/R_131_ H/H_131_  *P* value of HWE | 399 (21) 999 (51) 542 (28)  0.12 | 442 (23) 951 (49) 547 (28)  0.47 |
| FcγIIIB, n (%) NA2/NA2 NA1/NA2 NA1/NA1  *P* value of HWE | 816 (42) 872 (45) 252 (13)  0.43 | 844 (44) 848 (44) 248 (13)  0.13 |

F, phenylalanine; V, valine; R, arginine; H, histidine; HWE, Hardy-Weinberg equilibrium; NA, neutrophil antigen

# Supplementary Figures


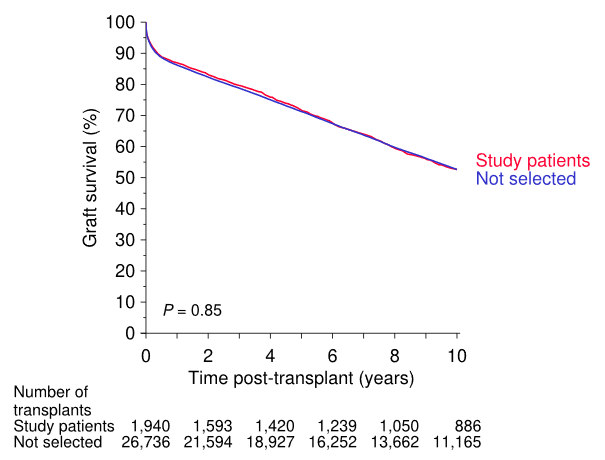


**Supplementary Figure 1.** Comparison of the graft survival between study patients and not selected patients who would have been eligible.


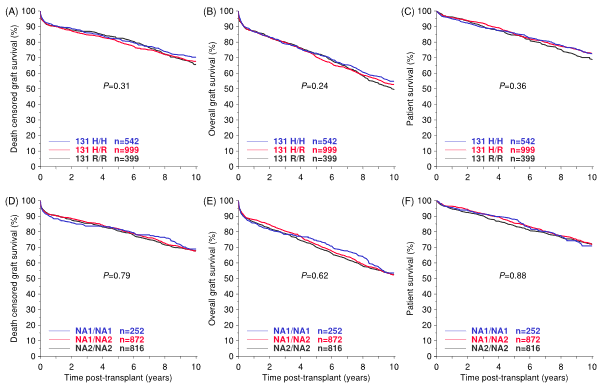


**Supplementary Figure 2.** Death-censored graft survival, overall graft survival and patient survival in relation to genotypic groups of *FCGR2A*-H/R131 and *FCGR3B*-NA1/NA2.


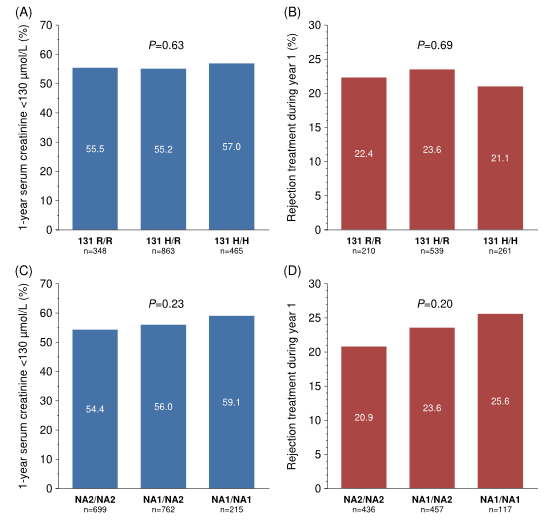


**Supplementary Figure 3.** Impact of FcγR polymorphisms FCGR2A-H/R131 and FCGR3B-NA1/NA2 on 1-year serum creatinine and on need for rejection treatment during the first post-transplant year.


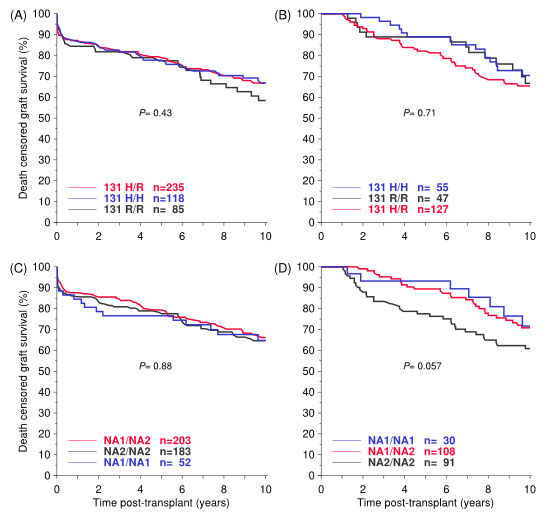


**Supplementary Figure 4.** Kaplan-Meier analysis of death-censored graft survival in 438 recipients with anti-HLA antibodies at the time of transplantation and 229 recipients with rejection treatment during the first post-transplant year, comparing the two homozygous groups and the heterozygous group of FCGR2A-H/R131 and FCGR3B-NA1/NA2.


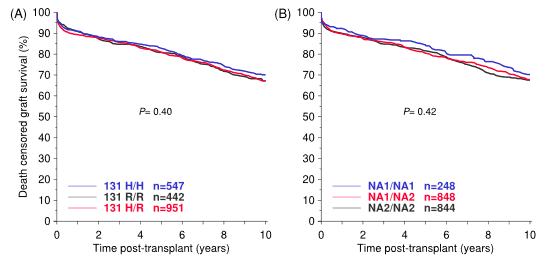


**Supplementary Figure 5.** Donor-derived FcγRIIA and FcγRIIIB polymorphisms and death-censored kidney allograft survival.
